# Aberrant methylation of *Polo-like kinase *CpG islands in *Plk4 *heterozygous mice

**DOI:** 10.1186/1471-2407-11-71

**Published:** 2011-02-15

**Authors:** Alejandra Ward, Alan Morettin, David Shum, John W Hudson

**Affiliations:** 1 University of Windsor, Department of Biological Sciences, 401 Sunset Avenue, Windsor, Ontario, N9B 3P4, Canada; 2 Windsor Regional Hospital, Metropolitan Campus, Department of Pathology, 1995 Lens Ave, Windsor, Ontario, N8W 1L9, Canada

## Abstract

**Background:**

Hepatocellular carcinoma (HCC), one of the most common cancers world-wide occurs twice as often in men compared to women. Predisposing conditions such as alcoholism, chronic viral hepatitis, aflatoxin B1 ingestion, and cirrhosis all contribute to the development of HCC.

**Methods:**

We used a combination of methylation specific PCR and bisulfite sequencing, qReal-Time PCR (qPCR), and Western blot analysis to examine epigenetic changes for the *Polo-like kinases *(*Plks*) during the development of hepatocellular carcinoma (HCC) in *Plk4 *heterozygous mice and murine embryonic fibroblasts (MEFs).

**Results:**

Here we report that the promoter methylation of *Plk4 *CpG islands increases with age, was more prevalent in males and that *Plk4 *epigenetic modification and subsequent downregulation of expression was associated with the development of HCC in *Plk4 *mutant mice. Interestingly, the opposite occurs with another Plk family member, *Plk1 *which was typically hypermethylated in normal liver tissue but became hypomethylated and upregulated in liver tumours. Furthermore, upon alcohol exposure murine embryonic fibroblasts exhibited increased *Plk4 *hypermethylation and downregulation along with increased centrosome numbers and multinucleation.

**Conclusions:**

These results suggest that aberrant *Plk *methylation is correlated with the development of HCC in mice.

## Background

The Polo-like kinases (Plks) are a highly conserved family of serine-threonine kinases, found from unicellular eukaryotic organisms to higher multicellular eukaryotes. The mammalian Plks (*Plk1-4*) have been shown to play major roles in cell cycle regulation, centrosome dynamics and the cellular response to stress. Furthermore, perturbations in individual Plk protein levels have been associated with malignancies. For example, high levels of Plk1 are indicative of a poor prognosis in esophageal, non-small cell lung cancer and oropharyngeal carcinomas [1,2] and have been observed in various forms of cancers including gastric, breast, ovarian, endometrial, gliomas, thyroid and melanomas [3]. In contrast, Plk3 is downregulated significantly in carcinomas of the lung, head and neck [4,5]. The *Plk2 *gene is downregulated in lymphomas and B-cell malignancies [6]. In the case of Plk4, over 50% of aged *Plk4 *heterozygous (*Plk4*^+/-^) mice develop tumours in comparison to only 3% of their wild-type littermates, the major site of tumour formation being the liver and lung [7]. In mice, *Plk4 *is haploinsufficient for tumour suppression, while in humans, loss of heterozygosity (LOH) for the *Plk4 *gene was found in 60% of a small sample of human hepatocellular carcinomas (HCC) cases[7]. The increased rate of tumourigenesis is likely related to the generation of aneuploidy, as altered Plk4 levels result in abnormal centrosome numbers [8], furthermore Plk4 may also play a key role in a DNA damage response pathway consistent with its phosphorylation of p53 [7], and Chk2 [9]. In general, overexpression of Plk1 is typically considered to be oncogenic in nature while the remaining Plks likely function as tumour suppressors.

Recently it has become evident that the hypermethylation of CpG islands of tumour-suppressor genes, histone modification and chromatin remodelling are common events in cancers (for review see [10]). Individual *Plk *gene epigenetic modifications associated with malignancy have previously been documented for *Plk2 *where its methylation-dependent silencing was detected at a high rate in B-cell malignancies and Burkitt's Lymphoma as well as in follicular lymphoma [11,12]. The correlation between the methylation status of the *Plks *and malignancy has not been studied in detail. In this regard, as noted below, we initially identified a gender disparity for the development of HCC in *Plk4*^*+/- *^mice. Previously, the development of HCC was attributed to haploinsufficiency for *Plk4 *rather than via loss of heterozygosity [7]. Given that there is accumulating evidence that epigenetic changes are a driving force in the development of HCC [13], we were interested in determining whether a relationship exists between individual *Plk *epigenetic modifications in the context of *Plk4 *haploinsufficiency and the development of HCC.

## Results and Discussion

### Plk methylation status in ageing mice and HCC samples

**Figure 1:**
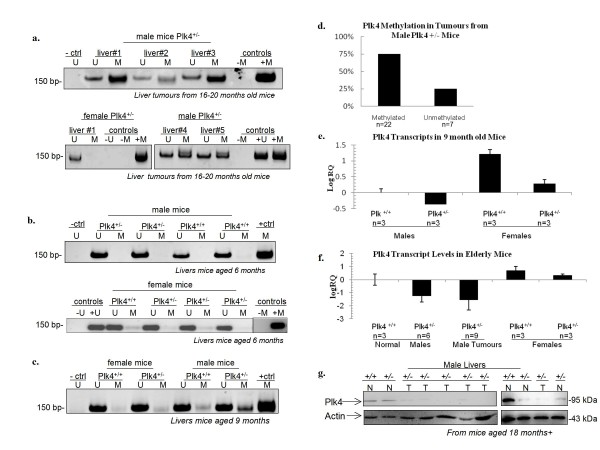
***Plk4 *CpG island methylation and expression levels in elderly *Plk4*^*+/-*^male mice and HCC samples**. Shown in each case (a-c) is a representative figure of typical results based on determination of *Plk4 *promoter methylation in 6-9 females and males for both *Plk4 *wild type and *Plk4*^+/- ^genotypes. (a) Methylation status of *Plk4 *promoter regions of genomic DNA extracted from liver tumours in *Plk4*^*+/- *^mice as determined by MSP. U = unmethylated, M = methylated. (b) *Plk4 *CpG island methylation of liver samples from mice aged 6 months and (c) 9 months. (d) Graphical representation summarizing percentage of *Plk4 *promoter methylation in liver tumours from 18-24 month old *Plk4*^*+/- *^male mice. (e) Relative levels of Plk4 transcripts as determined by qPCR. RQ values were normalized to the level of Plk4 transcripts in livers from 9 months old *Plk4*^*+/+ *^animals. The error bars represent the upper and lower limit of the standard error from the mean expression level (RQ). (f) Relative levels of Plk4 transcripts in liver tissue and tumours from elderly mice. (g) Level of Plk4 protein in liver tissue extracts as determined by Western blot analysis. Actin levels were used as a loading control. N = normal tissue, T = tumour tissue.

Sex specific predisposition to cancer may reflect the underlying effects of the methylation patterns of key cancer genes. While the mechanism remains unclear, gender disparity for HCC has previously been established in both humans and mice, where males are 3-5 times more likely to develop HCC than females [14,15]. Therefore, in the present study, we examined the rate of HCC in female and male *Plk4*^*+/- *^mice and found that in females the rate of HCC was approx 12% (n = 32) in comparison to 35% (n = 60) in male *Plk4*^+/- ^mice, indicative of a gender disparity for HCC development. An analysis of the mouse and human sequence databases revealed that three of four murine and all four human *Plk *genes have CpG rich regions at their 5' termini suggesting they may also be subject to regulation by promoter methylation. We examined the methylation status of the promoter region of the *Plk *genes from DNA extracted from aging mice for normal liver and liver tumours, and detected an increase in methylation status of the *Plk4 *gene in 22/29 tumours including 16/22 liver tumours studied in male mice (Figure 1a). Methylation status was confirmed *via *bisulfite sequencing of the *Plk4 *CpG island, in which 30-40% of the 38 CpG sites analyzed were methylated (Additional File 1). In contrast to the situation in males, we detected no *Plk4 *methylation in a small number of liver tumours found in females. Interestingly, at 6 months of age, no significant level of *Plk4 *CpG island methylation was detected in either male or female livers (Figure 1b). However, at 9 months of age and corresponding to our observed phenotype in aged mice, higher levels of *Plk4 *promoter methylation were detected in male mice in comparison to their female littermates (Figure 1c). In total, almost 80% of the HCC samples examined were methylated at *Plk4 *(Figure 1d). Similar disparities in the methylation status of individual genes associated with malignancy were previously found for RASSF1A in lung cancer, with males showing higher levels of methylation [16].

### The effect of aberrant Plk methylation on expression

Lower Plk4 levels likely play a role in malignancy by affecting genomic stability through a mechanism related to Plk4's role in centrosome duplication [8] and/or DNA damage pathways [17]. We therefore examined the levels of Plk4 transcripts and found that the levels were substantially lower in males *versus *female mice as early as 9 months of age (Figure 1e) and were greater than 10 fold lower in livers and liver tumours from aged *Plk4*^*+/- *^mice compared to wild type males and females and *Plk4*^*+/- *^females (Figure 1f). Similarly, Plk4 protein was also significantly reduced in tumours (Figure 1g). It is noted that, while livers from *Plk4*^+/- ^mice were grossly normal, they displayed variable amounts of Plk4 transcripts with an average that is significantly lower than that found in *Plk4*^+/+ ^mouse livers. Similarly, at the protein level, in *Plk4*^+/-^, we see varied amounts. It is noted that the *Plk4*^*+/- *^mice typically develop HCC 18-24 months on with some cases as early as 13 months. We propose that this likely reflects varying stages of progression towards the development of HCC; suggesting that reduced levels of Plk4 as a result of promoter methylation may precede the appearance of visible tumours. Low levels of Plk4 have been shown to result in the generation of mono-polar spindles and aneuploidy in both cell lines and tissues [7,8]. This exemplifies the possibility that epigenetic modifications may play a role in gender biases for malignancy and corresponds to our observation that epigenetic modifications of the *Plk4 *gene leads to further Plk4 downregulation, particularly in males.

**Figure 2:**
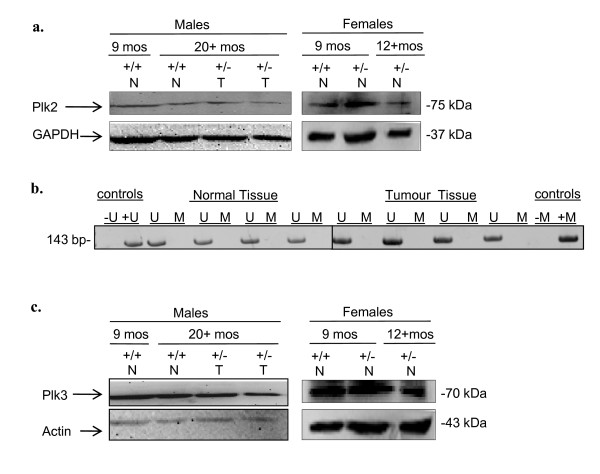
***Plk2 *methylation of CpG island and protein expression levels for Plk2 and Plk3 in relation to both age and gender in mice**. (a) Levels of Plk2 protein in liver tissue extracts as determined by Western blot analysis. (b) *Plk2 *CpG island methytlation status as determined by MSP analysis. (c) Levels of Plk3 protein in liver tissue extracts as determined by Western blot analysis. Shown are representative figures of 6-9 females and males for both *Plk4 *wild type and *Plk4*^+/- ^genotypes. N = normal, T = tumour. Note: we did not analyze *Plk3 *for methylation status as no CpG islands were detectable for the *Plk3 *gene with MethPrimer.

**Figure 3:**
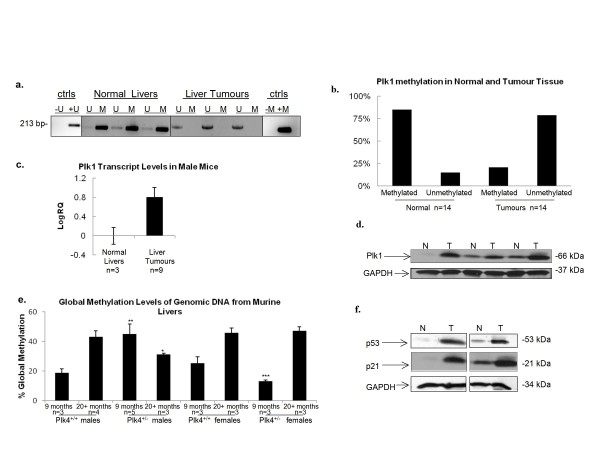
**Analysis of *Plk1 *CpG island methylation status and expression, global methylation, and expression levels for p53 and p21 in normal and liver tumour tissue samples**. (a) *Plk1 *CpG island methylation status for HCC samples compared to normal tissue as determined by MSP for aged-matched littermates. U = unmethylated, M = methylated. (b) Graphical representation summarizing the percentage of methylated Plk1 promoters in both normal liver tissue and tumours. (c) Plk1 transcript levels in normal liver and HCC samples as determined by qPCR. RQ values were normalized to the level of Plk1 transcripts in *Plk4*^*+/+ *^livers. The error bars represent the upper and lower limit of the standard error from the mean expression level (RQ) (d) Plk1 protein levels were examined by Western blot analysis. GAPDH protein levels were used as a loading control. N = normal, T = tumour. (e) The percent of global methylation of genomic DNA extracted from liver was determined by an ELISA assay specific for methylated DNA. (*p < 0.05), **p < 0.001, ***p < 0.05). The error bars represent the upper and lower limit of the standard error (f) p53 and p21 protein levels as detected by Western blot analysis. GAPDH levels were used as a loading control. N = normal, T = Tumour.

There is accumulating evidence that the Plk family of proteins often share the same targets or signalling pathways, thereby placing their substrates under tighter or opposing controls [18]. It was therefore of interest to determine whether *Plk4 *haploinsufficiency was also correlated with altered CpG island methylation and expression levels for the remaining *Plks*. Unlike the situation found in haematological malignancies [11], we found no significant change in either the methylation status or expression levels for *Plk2 *in tumours, aging mice or association with gender (Figure 2a-b). There were also no discernible changes in Plk3 protein levels (Figure 2c). Interestingly, the methylation status for *Plk1 *was opposite to that for *Plk4*. Normal tissue, regardless of age, showed methylation in the *Plk1 *promoter region in 80% of the samples tested (Figure 3a-b). However, *Plk1 *was found to be hypomethylated in 80% of HCC and other tumours found in *Plk4*^+/- ^mice (Figure 3a-b). Furthermore, this loss of promoter methylation corresponded to a large increase in Plk1 transcript levels (Figure 3c) and an increase in Plk1 protein level in HCC samples relative to normal liver tissue (Figure 3d). While the presence of increased Plk1 protein within tumours is by no means novel and is consistent with its potentially oncogenic role in malignancy, our findings indicate a novel mechanism for *Plk1 *regulation in that its expression may be influenced by its promoter methylation status, and, our results suggest that the transforming capacity of *Plk4 *heterozygosity may be linked to aberrant methylation of *Plk1 *and *Plk4*.

### Plk methylation status in human HCC samples

In order to determine if *Plk4 *methylation status is correlated with the development of HCC in humans, we also examined a limited number of human liver samples (See Additional File 2). We found that in normal human hepatic tissue the *Plk4 *promoter region was not methylated in samples taken from patients with no history of HCC. In the case of HCC samples, we detected *Plk4 *CpG island hypermethylation and downregulation of *Plk4 *transcript levels as well as barely detectable methylation of the *Plk1 *promoter region. In 3 of 6 samples we found that the corresponding Plk1 transcript levels were higher than in the normal control (Additional File 2e). We did not detect any changes for *Plk2 *and *Plk3 *promoter methylation (data not shown). Since we began this aspect of our study, Pellegrino *et al. *(2010) examined a large cohort of human HCC samples and reported *Plk2-3 *downregulation in human hepatocellular carcinoma correlated with either promoter hypermethylation and/or loss of heterozygosity at the *Plk2-3 *loci [19]. In the case of *Plk4*, many of the samples displayed loss of heterozygosity with no methylation within the *Plk4 *promoter region. They did not report any analysis for the methylation status of *Plk1*. Their inability to detect methylation changes for *Plk4 *and ours for *Plks2-3 *may be a reflection of the use of different primers for methylation specific PCR (MSP) (Additional file 3) which samples a small subset of the potentially methylated residues within a CpG island. Together these results suggest that in general, epigenetic changes within the *Plks *may contribute to malignancy in humans.

### Global methylation status and p53 activity

In general, global hypermethylation increases with age; however, studies on aberrant methylation of genes associated with HCC, like in many other malignancies, are characterized by an overall general increase in global hypomethylation along with increased rates of hypermethylation of tumour suppressors [20]. We employed an ELISA-based assay (Epigentek) in order to quantitatively measure genomic methylation. Interestingly, we found no significant difference between the 9 month old wild type males and age-matched wild type and *Plk4*^*+/- *^females (Figure 3e). However, consistent with what has been shown with age progression, we found an overall increase in the global methylation of genomic DNA in wild type male mice and both *Plk4 *wild type and heterozygous female mice from 9 to 20 months. In contrast, there was a decrease in global methylation in *Plk4*^*+/- *^male mice over the same time period (*p < 0.05). Furthermore, significantly higher levels of global methylation were found in young *Plk4*^+/- ^male mice compared to their wild type littermates (**p < 0.001), while the opposite is true for the *Plk4*^+/- ^female mice, where they had significantly lower levels of global methylation compared to young wild type females (***p < 0.05). Although, as the females age, both genotypes have similar levels of global methylation. These results suggest that there is an interplay between gender and *Plk4 *haploinsufficiency that affects global methylation in liver tissue.

p53 has also been found to be an upstream negative regulator of *Plk4 via *histone deactylation (HDAC) [21]. We therefore examined p53 levels in normal and tumour tissue and found that both p53 and p21 were upregulated in tumour tissue compared to the normal tissue (Figure 3f). p53 is also a substrate for Plk4 [22] and p53 levels/activity are upregulated as a result of haploinsufficiency in MEFs [17]. These observations suggest that increased p53 levels/activity, a consequence of *Plk4 *haploinsufficiency, may also contribute to repressive chromosome structure and the reduced transcript profiles seen in aged and tumourigenic *Plk4*^+/- ^mice.

### The effect of chronic alcohol exposure on Plk4 methylation status in MEFs

Alcohol has become an emerging environmental player in the modification of the epigenome [23]. In humans, chronic alcoholism has been shown to increase availability of blood homocysteines, which in turn modify s-adenosyl methyltransferase (MATs) levels, an enzyme responsible for the transfer of methyl groups to DNA. Furthermore, these patients showed a significant increase in global DNA methylation by up to 10% [24]. There is increasing evidence that alcohol consumption, a known risk for the development of HCC, can increase the methylation status of promoters with a subsequent decrease in gene expression [24-26]. In liver cells, the presence of alcohol results in an increase in the formation of reactive oxygen species, which are in turn responsible for hepatocyte damage, cellular apoptosis, and the tumour promoting effect of ethanol [27]. Interestingly, we have preliminary evidence of increased *Plk4 *methylation in human cirrhotic livers with no evidence of viral infection (see Additional File 2). This, coupled with the associated correlation between alcoholism and HCC development led us to examine the methylation status and expression of the individual *Plks *in a cell-based model of chronic ethanol exposure.

**Figure 4:**
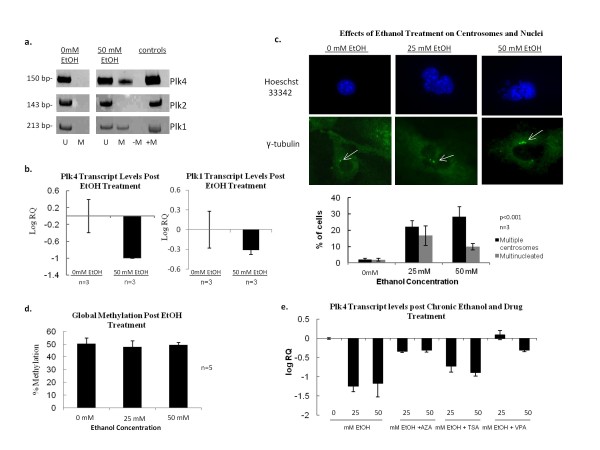
**The effect of chronic ethanol exposure on murine embryonic fibroblasts (MEFs)**. (a) MEFs were exposed to ethanol for a period of 7 days at which time the methylation status of individual *Plk *CpG islands was determined by MSP analysis. U = unmethylated, M = methylated. (b) Plk4 and Plk1 transcript levels in *Plk4 *wild type (*Plk4*^*+/+*^) MEFs were determined after 7 days of ethanol exposure by qPCR. RQ values were normalized to the level of transcript found in untreated control MEFs. Standard error was calculated based on the minimum and maximum values from the mean expression levels (RQ) (c) Immunofluorescence analysis Plk4^+/+ ^MEFs exposed to 25 mM and 50 mM ethanol for a period of 7 days. Centrosomes were detected by γ-tubulin staining and DNA by Hoechst staining. A graphical representation of cells exhibiting multiple centrosomes and multinucleation is underneath. Shown are the results of three independent experiments in which more than 200 cells were analyzed each time for each condition. Error bars indicate standard error. (d) Global methylation analysis of genomic DNA from MEFs after 7 days alcohol exposure as determined by MSP analysis of B1 elements. The error bars represent the upper and lower limit of the standard error from the mean. (e) Plk4 transcript levels as determined by qPCR of MEFs exposed to ethanol for 7 days in the presence of 5-aza-2'deoxycytidine (AZA), trichostatinA (TSA) and valproic acid (VPA). RQ values were normalized to the level of Plk4 transcript in untreated control MEFs. The error bars represent the upper and lower limit of the standard error from the mean expression level (RQ)

When wild type MEFs were exposed to a 25-50 mM dose of alcohol for 7 days, we found increased *Plk4 *promoter methylation and a significant decrease in corresponding Plk4 transcript levels (Figure 4a-b). (Note that in MEFs there was no methylation detected for the *Plks *pre-treatment). We also observed an increase in *Plk1 *promoter methylation although in this case the change in expression was not significant, displaying a large degree of variation. Furthermore, we found a large increase in the proportion of cells containing multiple centrosomes or multinucleation (Figure 4c), phenotypes correlated with reduced Plk4 levels in *Plk4*^*+/- *^mice [7]. Additionally, this observation mimicked the effect of lower Plk4 levels evident in *Plk4*^*+/- *^MEFs, which display increased centrosome numbers and ploidy with passaging [28,29]. Unexpectedly, in contrast to the situation found *in vivo *for chronic alcohol exposure [24-26], we found no evidence for increased global hypomethylation in MEFs (Figure 4d). However, these results do suggest that in MEFs that the *Plk4 *promoter may be a target for regulation by methylation in response to metabolic stress. This idea is supported by the fact that chronic alcohol exposure of MEFs has been shown to increase levels of reactive oxygen species (ROS) [30], as well as increase levels of p53 and p53 downstream targets such as p21 [31]. Interestingly, consistent with this p53 has been shown to indirectly repress Plk4 expression *via *HDAC in response to stress [22]. Additionally, while ROS have generally been shown to induce global hypomethylation [32], there is increasing evidence that they may also induce promoter hypermethylation. For example, both the E-cadherin and catalase promoters have been shown to become methylated post ROS exposure [33,34]. This is an area for future consideration.

### The effect of concurrent drug treatment on MEFs chronically exposed to alcohol

Unlike mutations or deletions that lead to the aberrant expression of tumour suppressor genes, epigenetic modifications, like DNA methylation, are reversible *via *the use of hypomethylating drugs that inhibit DNA methyltransferase activity and/or inhibit HDACs [35]. Concurrent alcohol and epigenetic drug treatments revealed that 5-aza-2'-deoxycytidine, a DNA hypomethylator, and valproic acid, which has been shown to be an HDAC inhibitor, partially restored Plk4 transcript levels, while no significant differences were seen with trichostatin A (an HDAC inhibitor) treatment (Figure 4e).

Modification of the methylation status and corresponding expression levels of both Plk4 and Plk1 are likely contributing factors in the development of HCC in both mice and humans. This creates interesting possibilities in that epigenetic modifications are potentially reversible through the use of demethylating and HDAC inhibiting drugs as both prophylactic and therapeutic tools. This may lead to the development of novel treatment options for HCC.

## Conclusions

We determined that a gender disparity exists for the development of HCC in the *Plk4 *mouse model. This disparity was correlated with increased DNA methylation at the *Plk4 *locus and higher risk of developing hepatocelluar carcinomas in aged male *Plk4 *heterozygous mice as compared to female mice. In contrast, we discovered the opposite correlation for *Plk1 *where in normal liver tissue the *Plk1 *promoter is hypermethylated while in tumours, *Plk1 *CpG islands become hypomethylated and the gene upregulated. This represents a novel form of regulation for Plk1 that may have implications for its expression in other tumour types. Furthermore, we determined that chronic alcohol exposure, well known to be implicated in the development of cirrhosis leading to HCC, also leads to *Plk4 *promoter hypermethylation and downregulation, accompanied by defects in the control of centrosome numbers and by the occurrence of multinucleation in cells. Aberrant *Plk4 *methylation and expression in chronically exposed MEFs could be rescued by treatment with known hypomethylating and/or HDAC inhibiting drugs.

## Methods

### Methylation specific PCR and global methylation

DNA from tissues was extracted as follows: 20-60 mg of tissue was digested with Pro K at a concentration of 0.5 mg/mL for 48 hrs at 55°C, followed by phenol chloroform extraction. DNA from formalin fixed paraffin embedded tissue was isolated using the FFPE DNA isolation kit following manufacturer's instruction (Qiagen). DNA from cells was isolated by trypsinization for 5 minutes, neutralization with media, centrifugation at 100 *g *for 5 minutes, resuspension with 200 ul of media, followed by Pro K treatment (20 mg/mL). Bisulfite modification was performed as previously described by Herman et al. 1996 [36]. The DNA was further purified with a Wizard Mini DNA clean up kit (Promega), followed by desulfanation with 2M NaOH for 10 min and ethanol precipitation. MSP was performed after bisulfite treatment of DNA. Mouse fully methylated genomic DNA (NEB) was used a as a positive control when assessing murine *Plks*. Primers were designed *via *MethPrimer [37] within the CpG islands of each individual *Plk *gene (see Table 1). Global methylation levels for liver tissue were determined by the MethylFlash Methylated DNA Quantification Kit (Epigentek), an ELISA-based colourimetric assay. The assay was done according to the manufacturer's instructions, using 100 ng of genomic DNA. The Wallac Victor3 1420 multilabel counter was used to measure the assay at 450 nm. Relative quantification was determined by normalizing the readings to the positive control provided with the kit. In ethanol treated mouse embryonic fibroblasts global methylation was assessed by determining the methylation status of B1 elements with MSP as previously described by Jeong *et al. *2005 [38]. Briefly, there are 30, 000 copies of the 163 base pair element dispersed throughout the mouse genome. Each element contains 6 CpG dinucleotides. The methylation status of these elements is also responsive to DNA methyltransferase inhibitors like Azacytidine and therefore they are excellent indicators of global methylation. In order to determine the percentage of B1 element methylation densitometry was performed with analysis *via *the Syngene Gene tools version 3.07 software. Statistical analysis on the normalized results were performed with the Statsoft Statistica v7.0.61.0 and a one-way ANOVA t-test where p < 0.05 was significant.

**Table 1:** Mouse primer sequences

Target Gene	Sense Primer	Antisense Primer
Plk1 U	5'aca aac acc tct ttt ata tct aca tc 3'	5'tgg ttt gag tat tag ttg att ttg g 3'
Plk1 M	5'acg aac acc tct ttt ata tct acg tc 3'	5'gtt ggt tcg agt att agt cga ttt c 3'
Plk2 U	5' caa act tta ccc aaa acc tac tcac 3'	5'ata ggg tta gtt tgg atg ttt gtt t 3'
Plk2 M	5' aaa ctt tac cca aaa cct act cg 3'	5'ggt tag ttc gga cgt ttg ttc 3'
Plk4 U	5'cac act ctc cac ttc tta aaa aca a 3'	5' att tta tta tta gtg ttt gtg tta tgg 3'
Plk4 M	5'aca ctc tcc act tct taa aaa cga a 3'	5' aat tta tta tta gcg ttc gcg tta c 3'
B1 Element U	5'-taa cct caa act caa aaa tcc acc-3'	5'gtt ggg tgt agt ggt ata tat ttt taa ttt ta 3'
B1 Element M	5'ctcgaactcaaaaatccgcc 3'	5' gtc ggg cgt agt ggt ata tat ttt t 3'

### Tissue Samples

All murine samples were obtained from our breeding colony, with all protocols for animals approved by the University of Windsor Animal Care Committee according to the Canadian Council on Animal Care guidelines. *Plk4*^*+/- *^mutant mice on a 129Sv/CD1 background were obtained as described [28] and backcrossed with CD1 mice to establish a colony of *Plk4 *wild type and *Plk4 *heterozygous littermates. Mice were maintained under normal light cycle and on regular chow. All tissue samples were obtained from aged matched littermates. For murine hepatocellular carcinoma (HCC) samples, it is noted that *Plk4*^*+/- *^mice develop a high rate of liver and lung tumours by 18-24 months of age [7] and thus the analysis was performed on spontaneously occurring hepatocellular carcinomas.

### Cell lines

Mouse embryonic fibroblasts (MEFs) were harvested from *Plk4 *wild type CD1 mice at day 12.5 post coitum as described previously in [28] and cultured with DMEM supplemented with 20% FBS (Sigma), 1% penicillin G sodium 10,000 U/mL, streptomycin sulphate 10,000 ug/mL, and gentamycin 10 mg/mL.

### Western blot analysis

Protein from fresh tissue was extracted using the Trizol reagent (Invitrogen) according to manufacturer's provided protocols. Cell lysates were obtained from cells treated with buffer containing 50 mM Tris-HCl pH 7.4, 150 mM NaCl, 1 mM EDTA, 0.5% Triton X with EDTA free protease inhibitor cocktail tablets (Roche). Western blot analysis was performed using 20 ug of total protein. Primary antibodies were as follows: p53 (Sigma), Plk1 (Abcam), p21, Plk2, Plk3 (Santa Cruz), Plk4, GAPDH (Cell Signaling), and Actin (Sigma). Secondary antibodies were anti-rabbit (Amersham) and anti-mouse horseradish peroxidase (HRP) (Sigma).

### Analysis of gene expression

RNA was extracted from cells and tissues using the RNAeasy kit (Qiagen) according to manufacturer's recommendations. cDNA was generated using the "First Strand cDNA synthesis kit" according to the manufacturer's instructions. Quantitative real time PCRs (qPCR) were conducted in an ABI 7300 instrument using 250 ng of cDNA with TaqMan Gene Expression Assays (Applied Biosystems) for mouse *Plk1 *and *Plk4*. Rodent GAPDH probe was used as an internal control. Relative quantity (RQ) values were generated by the ABI 7300 system SDS software. The error bars represent the upper and lower limit of the standard error from the mean expression level (RQ) as analyzed by the SDS software. The error bars are calculated based on 95% confidence limits.

### Immunofluorescence

MEF cells were fixed in 3.7% paraformaldehyde and probed with a mouse γ-tubulin primary antibody (Sigma) followed by an anti-mouse alexa fluor 568 secondary antibody (Invitrogen). The cells were then briefly incubated in Hoescht 33342. Cells were analyzed with a Zeiss Axioskop 2 mot plus microscope and Northern Eclipse imaging software. Conditions for immunofluorescence were as described previously [28].

### Ethanol and drug treatments

Wild type MEFs were exposed to 25 mM or 50 mM ethanol per day for 7 days. Trichostatin A, 5 aza-2'-deoxycytidine, and valproic acid were administered concurrently at concentrations of 1 nM, 10 nM, and 0.5 mM respectively.

## Competing interests

The authors declare that they have no competing interests.

## Authors' contributions

AW carried out all the studies reported in the manuscript and was directly involved in the conception of experiments, analysis and writing of the manuscript. AM was involved in the isolation of MEFs and studies on *Plk *promoter methylation in human samples. DS acted as a consultant throughout these studies. JWH was involved in the conception and analysis of the entire study as well as the drafting of the manuscript. All authors read and approved the final manuscript.

## Pre-publication history

The pre-publication history for this paper can be accessed here:

http://www.biomedcentral.com/1471-2407/11/71/prepub

## Supplementary Material

Additional file 1 **Bisulfite sequencing PCR for the *Plk4 *promoter region in HCC**. The CG sites within the *Plk4 *promoter were sequenced in hepatocellular carcinoma cases and compared to a fully methylated control and normal wild type liver samples Click here for file

Additional file 2 **Profiling the methylation of the Polo-like kinases in human liver and HCC**. Human normal liver and tumour samples were assessed in order to determine the methylation status of the individual *Plks *and to determine the transcript levels of *Plk1 *and *Plk4* Click here for file

Additional file 3 **Materials and methods for experiments conducted in additional files **1 and 2 Contains the description of BSP, MSP, qPCR, and a table with all the human primers used. Click here for file
