# Supplementary material for: Aberrant methylation of Polo-like kinase CpG islands in Plk4 heterozygous mice
Source: BMC Cancer. 2011 Feb 15;11:71. doi: 10.1186/1471-2407-11-71 (PMC3047422; doi:10.1186/1471-2407-11-71)
Supplement: Additional file 1 — Bisulfite sequencing PCR for the Plk4 promoter region in HCC. The CG sites within the Plk4 promoter were sequenced in hepatocellular carcinoma cases and compared to a fully methylated control and normal wild type liver samples [file 1471-2407-11-71-S1.PPT]

## Slide 1
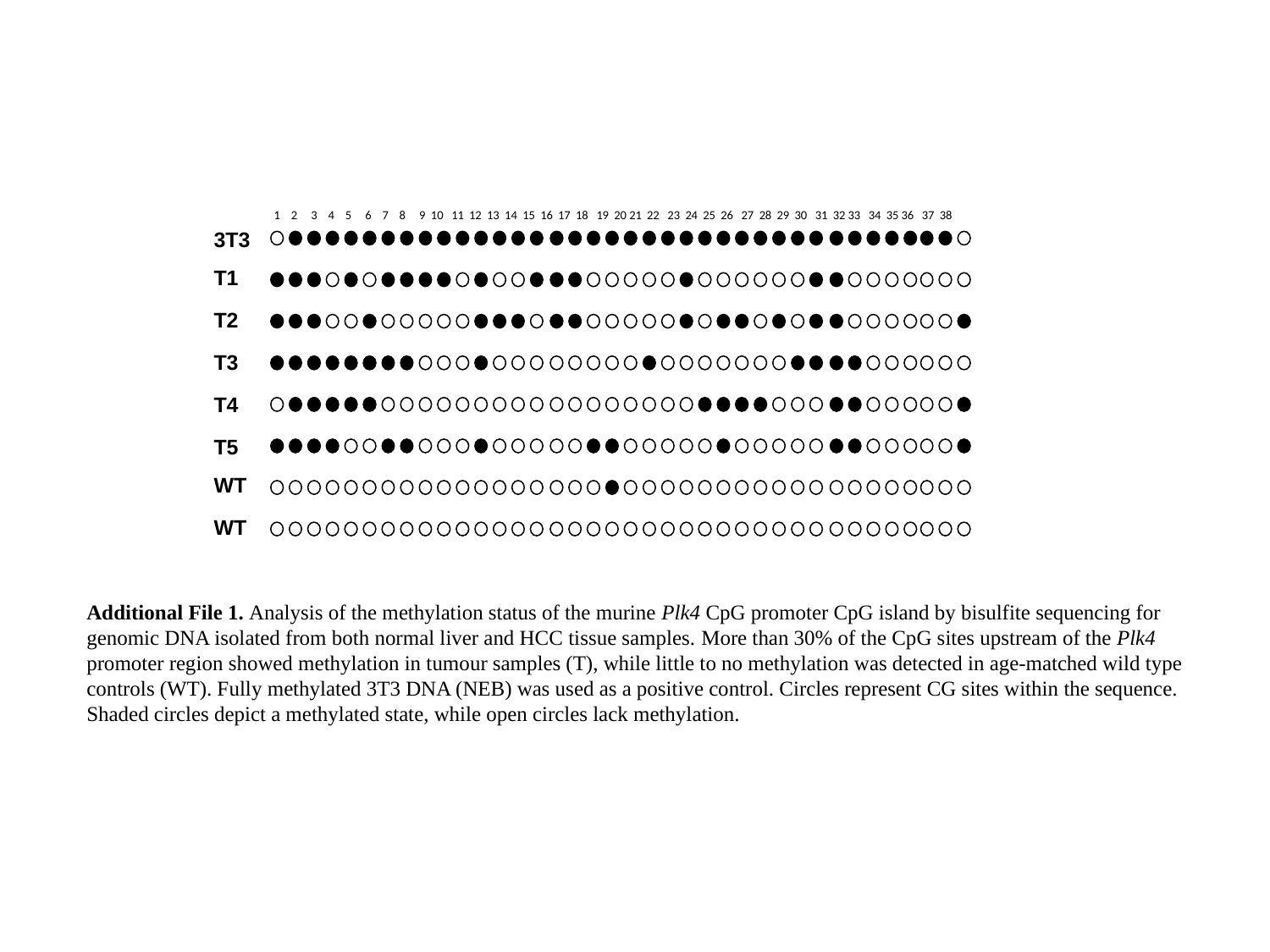

1 2 3 4 5 6 7 8 9 10 11 12 13 14 15 16 17 18 19 20 21 22 23 24 25 26 27 28 29 30 31 32 33 34 35 36 37 38
3T3
T1
T2
T3
T4
T5
WT
WT
Additional File 1. Analysis of the methylation status of the murine Plk4 CpG promoter CpG island by bisulfite sequencing for genomic DNA isolated from both normal liver and HCC tissue samples. More than 30% of the CpG sites upstream of the Plk4 promoter region showed methylation in tumour samples (T), while little to no methylation was detected in age-matched wild type controls (WT). Fully methylated 3T3 DNA (NEB) was used as a positive control. Circles represent CG sites within the sequence. Shaded circles depict a methylated state, while open circles lack methylation.
