# Supplementary material for: Aberrant methylation of Polo-like kinase CpG islands in Plk4 heterozygous mice
Source: BMC Cancer. 2011 Feb 15;11:71. doi: 10.1186/1471-2407-11-71 (PMC3047422; doi:10.1186/1471-2407-11-71)
Supplement: Additional file 2 — Profiling the methylation of the Polo-like kinases in human liver and HCC. Human normal liver and tumour samples were assessed in order to determine the methylation status of the individual Plks and to determine the transcript levels of Plk1 and Plk4 [file 1471-2407-11-71-S2.PPT]

## Slide 1
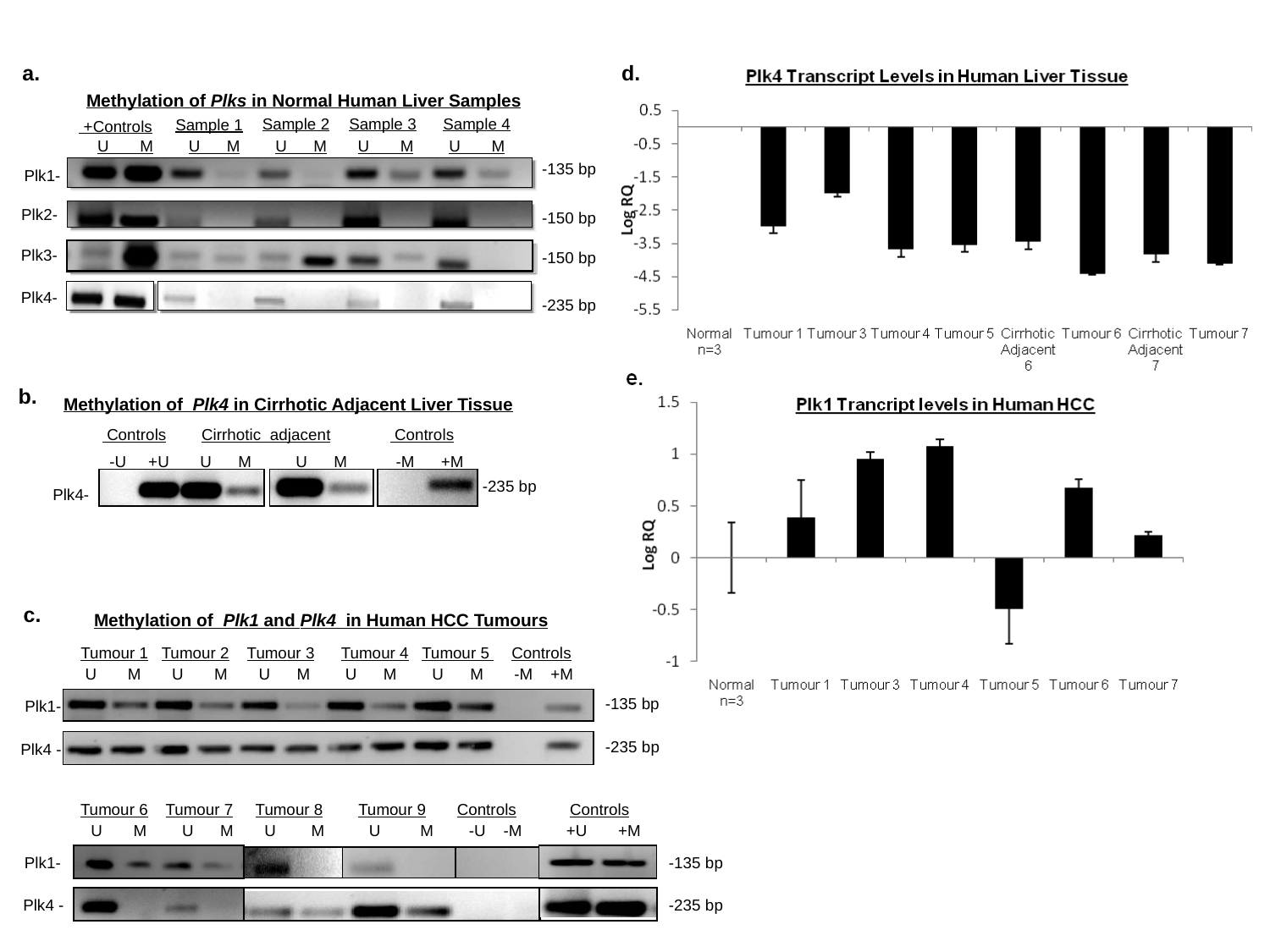

a.
Methylation of Plks in Normal Human Liver Samples
Sample 2
Sample 3
Sample 4
Sample 1
 +Controls
 U M U M U M U M U M
Plk1-
Plk2-
Plk3-
Plk4-
-135 bp
-150 bp
-150 bp
-235 bp
d.
b.
 Controls
Cirrhotic adjacent
 Controls
Plk4-
 -U +U U M U M -M +M
-235 bp
Methylation of Plk4 in Cirrhotic Adjacent Liver Tissue
c.
Methylation of Plk1 and Plk4 in Human HCC Tumours
 Tumour 1 Tumour 2 Tumour 3 Tumour 4 Tumour 5 Controls
 U M U M U M U M U M -M +M
Plk1-
Plk4 -
-135 bp
-235 bp
 Tumour 6 Tumour 7 Tumour 8 Tumour 9 Controls Controls
 U M U M U M U M -U -M +U +M
Plk1-
-135 bp
-235 bp
Plk4 -

## Slide 2
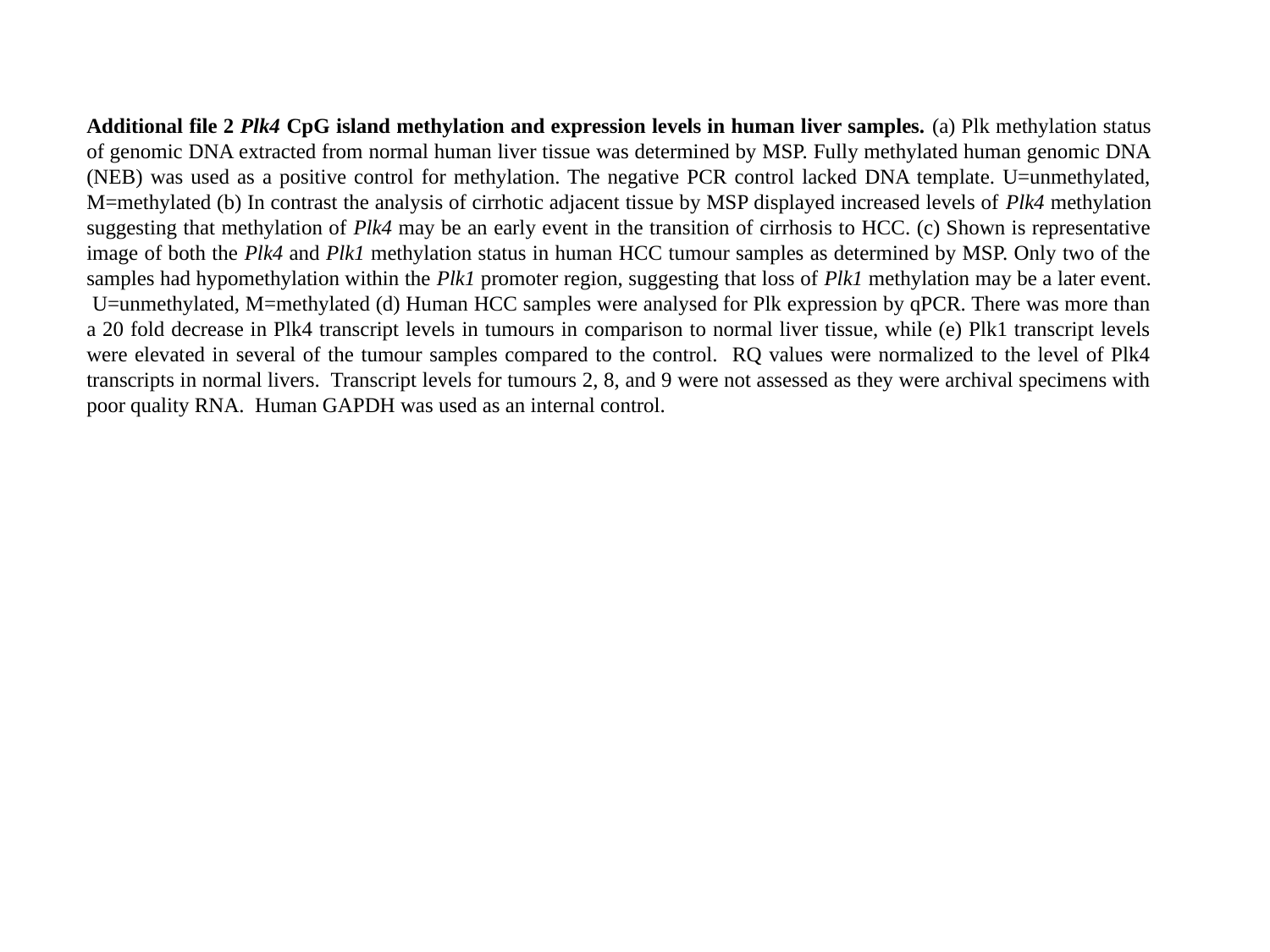

Additional file 2 Plk4 CpG island methylation and expression levels in human liver samples. (a) Plk methylation status of genomic DNA extracted from normal human liver tissue was determined by MSP. Fully methylated human genomic DNA (NEB) was used as a positive control for methylation. The negative PCR control lacked DNA template. U=unmethylated, M=methylated (b) In contrast the analysis of cirrhotic adjacent tissue by MSP displayed increased levels of Plk4 methylation suggesting that methylation of Plk4 may be an early event in the transition of cirrhosis to HCC. (c) Shown is representative image of both the Plk4 and Plk1 methylation status in human HCC tumour samples as determined by MSP. Only two of the samples had hypomethylation within the Plk1 promoter region, suggesting that loss of Plk1 methylation may be a later event. U=unmethylated, M=methylated (d) Human HCC samples were analysed for Plk expression by qPCR. There was more than a 20 fold decrease in Plk4 transcript levels in tumours in comparison to normal liver tissue, while (e) Plk1 transcript levels were elevated in several of the tumour samples compared to the control. RQ values were normalized to the level of Plk4 transcripts in normal livers. Transcript levels for tumours 2, 8, and 9 were not assessed as they were archival specimens with poor quality RNA. Human GAPDH was used as an internal control.
