# Supplementary material for: Aberrant methylation of Polo-like kinase CpG islands in Plk4 heterozygous mice
Source: BMC Cancer. 2011 Feb 15;11:71. doi: 10.1186/1471-2407-11-71 (PMC3047422; doi:10.1186/1471-2407-11-71)
Supplement: Additional file 3 — Materials and methods for experiments conducted in additional files 1 and 2 Contains the description of BSP, MSP, qPCR, and a table with all the human primers used. [file 1471-2407-11-71-S3.DOC]

**Additional File 3**

**Supplementary Materials and Methods:**

*Human Primers*

Primers for methylation specific PCR were determined by MethPrimer and are as shown in Supplementary Tables 1 (Human).

*Human HCC samples*

Human HCC samples were obtained from both the Ontario Tumour Bank and the Alberta Tumour Bank. All protocols were approved by the individual tumour banks and the University of Windsor Research Ethics Board.

*Analysis of gene expression*

RNA was extracted from tissues using the RNAeasy kit (Qiagen) according to manufacturer’s recommendations. cDNA was synthesized using the “First Strand cDNA synthesis kit” (Invitrogen) according to the manufacturer’s instructions. Quantitative real time PCRs (qPCR) were conducted in an ABI 7300 instrument using 250 ng of cDNA with TaqMan Gene Expression Assays (Applied Biosystems) for human *Plk1 and* *Plk4*. Human GAPDH probe was used as an internal control. Relative quantity (RQ) values and error bars were generated by the ABI 7300 system SDS software. The error bars represent the upper and lower limit of the standard error from the mean expression level as analyzed by the SDS software with the ABI 7300 instrument. The error bars are calculated based on 95% confidence limits.

*Bisulfite Sequencing PCR*

Bisulfite sequencing PCR (BSP) for *Plk4* CpG islands was done by bisulfite converting 2ug of genomic DNA, as described previously (30) followed by PCR amplification of the *Plk4* promoter region with BSP Primers as follows: 5’GCGCT ACGGTCAGTCGTACACTGACC3’, 5’GAGGTTGAGGTTTAGTTTGGTT3’, 5’ AAAT T TTCTAAACTCCCTCCCT 3’. BSP primers were determined with the methyl-primer software package (ABI). PCR products were gel purified using the Qiaquick gel extraction kit (Qiagen) and analysed by direct sequencing or subcloned into the p-GEM-T-easy cloning vector (Promega). Sequence analysis was performed using the ABI 3730 with sequencing chemistry BDT version 3.1.We initially performed direct sequencing of 5 independent PCR amplifications for each tumour sample to identify methylated CpG sites in individual tumours. The sites were confirmed by a cloning method in which 10 subclones were picked for sequencing per tumour sample. Individual CpG sites were considered to be positive for methylation where a minimum of 60% of the subclones displayed a methylated nucleotide.

**Supplementary Table 1.**

Human primer sequences for MSP:

| Gene | Sense Primer | Antisense Primer |
| --- | --- | --- |
| Plk1 U | 5’ tag tga ttg tag gga agt tgg t 3’ | 5’aca taa atc cac taa aac ctc c 3’ |
| Plk1 M | 5’ gta aat cca cta aaa cct cc 3’ | 5’ gta gtg att gta ggg aag ttg 3’ |
| Plk2 U | 5’cac ccc aca acc aac caa aca 3’ | 5’ gga tgg ttt tga agg ttt ttt t 3’ |
| Plk2 M | 5’ ccc acg acc gac cga acg c 3’ | 5’ acg gtt ttg aag gtt ttt tcg c 3’ |
| Plk3 U | 5’agt aaa ttt agg tag tgt tat 3’ | 5’ aaa ccc aac caa aaa aac a 3’ |
| Plk3 M | 5’aat tta ggt agc gtt acg cgc 3’ | 5’ccg acc gaa aaa acg aac gc 3’ |
| Plk4 U | 5’cca aac tct aac cta aat tct cca a 3’ | 5’ att att agt tta gtt tgg atg gta agt gg |
| Plk4 M | 5’caa act cta acc taa att ctc cga a 3’ | 5’tat tag ttt agt tcg gac ggt aag c 3’ |
